# The expression of C1 inhibitor (C1INH) in macrophages is upregulated by retinal pigment epithelial cells – implication in subretinal immune privilege in the aging eye

**DOI:** 10.18632/aging.101474

**Published:** 2018-06-13

**Authors:** Chang Luo, Jiawu Zhao, Mei Chen, Heping Xu

**Affiliations:** 1 Centre for Experimental Medicine, School of Medicine, Dentistry & Biomedical Science, Queen’s University Belfast, Belfast, UK; 2 AIER Eye Institute, Changsha, China; 3 AIER School of Ophthalmology, Central South University, Changsha, China

**Keywords:** macrophages, retinal pigment epithelial cells, complement, aging, subretinal immune privilege

## Abstract

Age-related para-inflammation in the retina-choroidal interface is featured by low-levels of complement activation and subretinal macrophage accumulation. This study aimed to understand how complement expression in macrophages is regulated by retinal pigment epithelium (RPE). Bone marrow-derived macrophages (BMDMs) and RPE cells were cultured from 8-10 weeks old C57BL/6J mice. The BMDMs were co-cultured with normal RPE, or oxidized photoreceptor outer segment (oxPOS) or TNF-α pre-treated RPE, or apoptotic RPE, or RPE-choroid eyecups. Macrophages were then isolated and processed for real-time RT-PCR. The expression of complement inhibitor C1INH in BMDMs was significantly upregulated by RPE and RPE-choroid eyecups. The eyecups also upregulated CFH, CD59a, and Crry in BMDMs. oxPOS pre-treated RPE upregulated C1qb but down-regulated C3 expression in BMDMs. TNF-α pre-treated RPE enhanced C1INH and CFB expression. When BMDMs were treated with apoptotic RPE, the expression of C1qb, CFH, and CD59a was reduced, whereas the expression of C3, CFB and C1INH was increased. Our results suggest that RPE can modulate macrophages complement expression at the retina-choroidal interface even under aging or oxidative conditions. However, during inflammation, they may promote the alternative pathway of complement activation through down-regulating CFH and CD59a and upregulating CFB and C3.

## Introduction

The neuronal retina is segregated from the systemic immune system by the blood retina barriers (BRB) and is considered as an immune privileged tissue. In addition to the physical barrier, the immune suppressive microenvironment of the eye is also critical for retinal immune privilege [1–3]. The immune privilege reduces the likelihood of intraocular inflammation, thus protects the neuroretina from inflammation-mediated damage. Despite the lack of systemic immune surveillance, the retina is well-protected by its own innate immune defence system, including innate immune cells (e.g., microglia and perivascular macrophages) and the complement system [4]. The pathophysiology of retinal innate immune cells, particularly microglia has been well-studied and the subject has been reviewed extensively recently (see review article [5–7]). However, how the complement system protects the retina remains poorly defined.

The complement system constitutes over 30 proteins and protein fragments. Most of the complement proteins are known to be synthesized in the liver and released into circulation as inactive precursors. Recent evidence suggests that complement components are also produced outside the liver by tissue cells [8]. Previous studies from our group and others have shown that the retina and retinal pigment epithelium (RPE)/choroidal tissue express a variety of complement components [9–11], and microglia and RPE are the major sources of local complement expression [9,12]. During aging, the expression of complement proteins or fragments is increased in the retina, particularly at the retina-choroid interface [4,11,13,14]. Together with a low level of microglial activation, the increased immune reactivity in the aging retina (also known as para-inflammation [14,15]) maintains retinal homeostasis and functionality [14].

**Figure 1:**
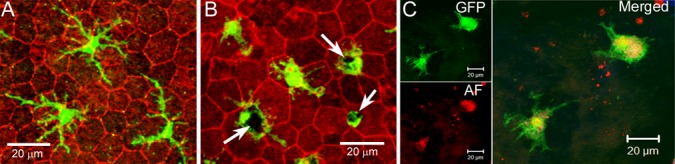
**Subretinal macrophages in the aging eye.** RPE/choroidal flatmount from 18 months (**A**) and 24 months (**B**) old CX3CR1^gfp/+^ mice were stained with phalloidin and imaged by confocal microscopy. Arrows - pigmented debris in subretinal macrophages. (**C**), RPE/choroidal flatmount from a 24 month old CX3CR1^gfp/+^ mouse was imaged by confocal microscopy. Green – GFP+ subretinal macrophages; Red – autofluorescence (AF).

The retina-choroid interface undergoes progressive changes during aging. For example, RPE cells increase in size and become multinucleate [16], phagocytes (microglia and macrophages) accumulate in the subretinal space (Figure 1) [17–19]. RPE cells and macrophages can both produce complement proteins [9,12], and they are the major sources of complement expression at the retina-choroid interface in the normal aging eye. How their complement expression is regulated during aging and at disease conditions is an important question. We have shown previously that complement expression in RPE cells is regulated by active macrophages [12]. We have also shown that complement expression in macrophages is regulated by cytokines presented at the microenvironment [20]. Macrophages are in close contact with RPE cells at the subretinal space in the aging eye (Figure 1). The aim of this study was, therefore, to investigate how complement expression in macrophages is regulated by RPE cells.

Under normal physiological conditions, RPE cells can suppress macrophage activation through pigment epithelial-derived factor (PEDF) [21–23] and α-melanocyte stimulating hormone (α-MSH) [24]; whereas, under disease conditions, particularly when RPE cells undergo apoptotic death, the dead RPE cells can induce an angiogenic phenotype in macrophages [25]. Although substantial RPE death unlikely occurs in the normal aging eye, the cells are subjected to chronic oxidative stress and are constantly undergoing morphological remodeling [16]. The ability of RPE cells to modulate macrophage function in the subretinal space may depend on the condition of RPE cells. Indeed, subretinal macrophages in the healthy adult eyes (6 -12 months old) often have a small soma and long- fine-dendrites (Fig. 1A), whereas the cells in the aging eye (20 – 27 months) have a large cell body that often contains pigmented debris (Fig. 1B) [15], and they are autofluorescent (Fig. 1C) [17]. This suggests that they are active phagocytizing debris released by stressed RPE cells. In this study, we investigated the effect of different types of RPE cells in macrophage complement expression, including normal RPE cells, oxidized photoreceptor outer segments (oxPOS) pre-treated or TNF-α pre-treated RPE cells and apoptotic RPE cells.

## RESULTS

### The effects of normal RPE cells on BMDM complement gene expression

**Figure 2:**
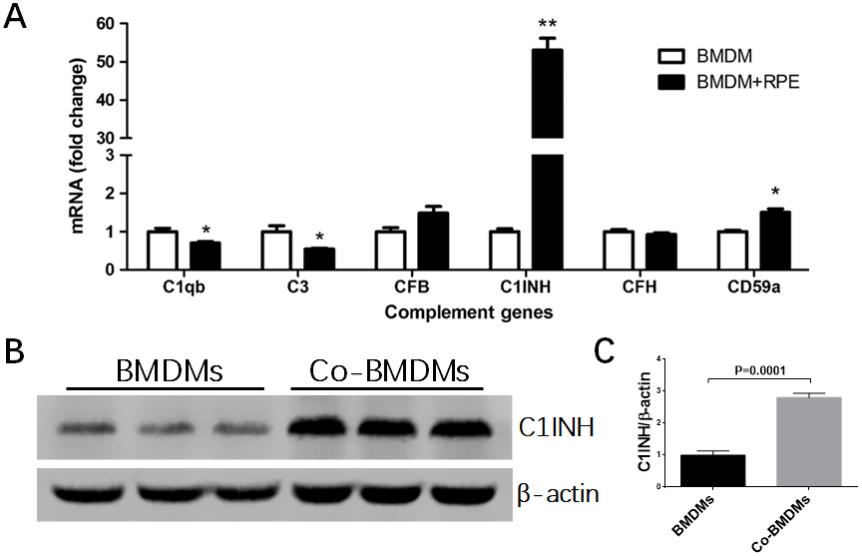
**The effects of normal RPE cell on BMDM complement expression.** BMDMs from C57BL/6J mice were co-cultured with primary mouse RPE cells for 7h (**A**) or 24h (**B**). Macrophages were then isolated by CD11b^+^ MACS kit and processed for real-time RT-PCR analysis of complement genes (**A**) and western blot analysis of C1INH protein expression (**B**). Fold change of C1INH protein expression by BMDMs after co-culture was analyzed by ImageJ (**C**). Mean ± SEM, n =3; *, P< 0.05; **, P < 0.01 compared to naïve BMDM alone, Unpaired Student t test.

Previously we have shown that macrophages express a variety of complement components and regulatory genes [20]. In this study, we selectively investigated complement genes that were expressed at reasonable levels in macrophages in our previous study, including *c1qb* and *c1inh* of the classical pathway, *cfb* and *cfh* of the alternative pathway, and *c3* and *cd59a* of the terminal pathway. When BMDMs were co-cultured with normal RPE cells, the expression of C1qb and C3 mRNA was significantly reduced (Figure 2), whereas the mRNA expression of CD59a, particularly C1INH was markedly increased (1.85-fold and 53.07-fold respectively) (Figure 2A). The upregulation of C1INH was further confirmed at protein level by Western Blot (Figure 2B, 2C). The expression of CFB and CFH was not affected. C1INH and CD59a negatively regulate complement activation. Our result suggests that under normal physiological conditions, RPE cells may suppress complement activation at the retina—choroid interface by modulating subretinal macrophage complement expression.

### The effects of oxPOS pre-treated RPE cells on BMDM complement gene expression

**Figure 3:**
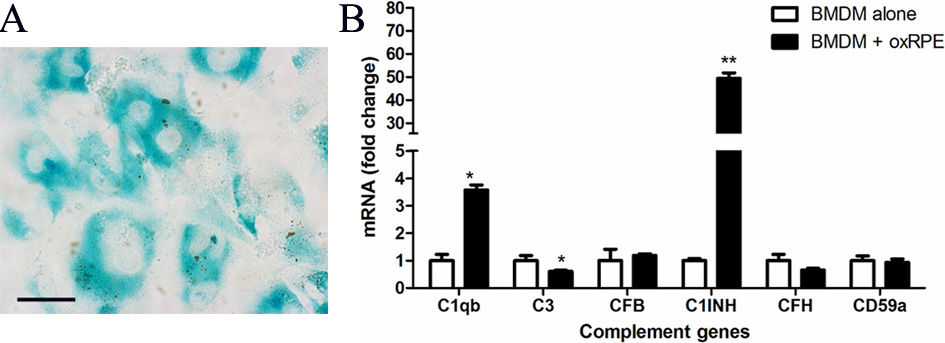
**The effects of oxidized POS treated-RPE cell on BMDM complement gene expression.** RPE cells were treated with oxidized photoreceptor outer segments (oxPOS) for 24h. oxPOS were then removed from the culture. (**A**) β-galactosidase expression in ox-POS-treated RPE cells. (**B**) The oxPOS pre-treated RPE cells were co-cultured with naïve BMDMs for 7h. Macrophages were isolated and processed for real-time RT-PCR analysis of complement genes. Mean ± SEM, n =3; *, P< 0.05; **, P < 0.01 compared to naïve BMDM alone, Unpaired Student t test.

Our recent work suggests that oxidized POS (oxPOS) suppresses RPE proliferation and induces multinucleation, a phenotype that is similar to RPE cells in the aging eye [16]. In this study, we further found that oxPOS treatment induced β-galactosidase expression in RPE cells (Figure 3A), an indicative of cell senescence. When BMDMs were co-cultured with oxPOS pre-treated RPE, the expression of C1qb increased by more than 3-fold. The expression of C1INH remained at high levels (49.36-fold increment) compared with untreated BMDMs, whereas the expression of C3 was significantly decreased (Figure 3B). The expression of other genes, including CFB, CFH, and CD59a was not affected (Figure 3B). C1q is involved not only in the CP complement activation, but also in phagocytosis [26]. Our results suggest that, RPE cells in the aging eye may suppress complement activation through macrophage related C1INH and promote subretinal macrophage phagocytosis by enhancing C1q expression.

### The effects of TNF-α pre-treated RPE cells on BMDM complement gene expression

**Figure 4:**
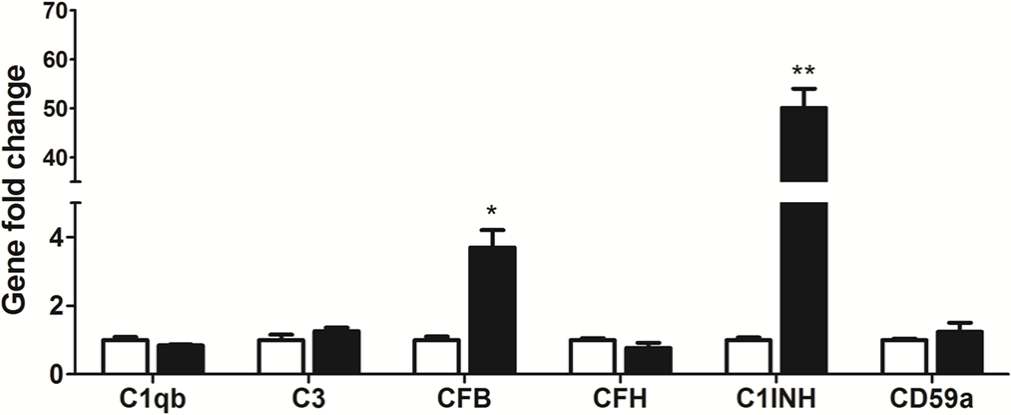
**The effects of TNF-α treated-RPE cell on BMDM complement gene expression.** RPE cells were treated with TNF-α for 16h. Naïve BMDMs were then co-cultured with TNF-α pre-treated RPE cells for 7h. Macrophages were isolated and processed for real-time RT-PCR analysis of complement genes. Mean ± SEM, n =3; *, P< 0.05; **, P < 0.01 compared to naïve BMDM alone, Unpaired Student t test.

TNF-α is one of the key inflammatory mediators in the inflamed eye e.g., uveoretinitis [27–29]. When BMDMs were co-cultured with TNF-α pre-treated RPE cells, the expression of CFB and C1INH was increased by 3.7-fold and 50.1-fold respectively (Figure 4), whereas other complement component genes, including C1qb, C3, CFH and CD59a remained unchanged (Figure 4). The result suggests that under inflammatory conditions, RPE cells may convert macrophages into a phenotype that can promote complement activation through the alternative but not the classical or MBL pathway.

### The effects of apoptotic RPE cells on BMDM complement gene expression

**Figure 5:**
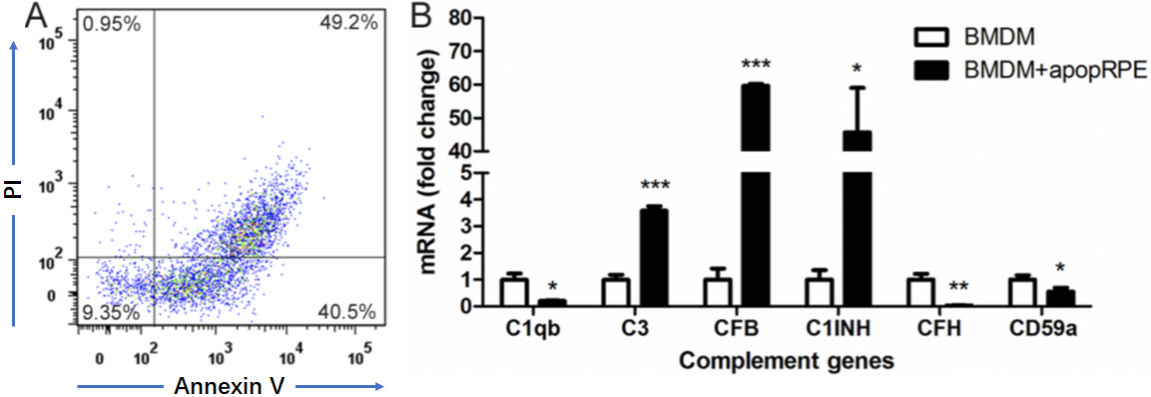
**The effects of apoptotic RPE cell on BMDM complement gene expression.** Primary mouse RPE cells were treated with H_2_O_2_ in serum-free DMEM overnight. Apoptotic RPE cells were confirmed by Propidium iodide (PI) and Annexin V staining (**A**). The apoptotic RPE cells were then incubated with naïve BMDMs for 7h. Macrophages were isolated and processed for real-time RT-PCR analysis of complement genes (**B**). Mean ± SEM, n =3; *, P< 0.05; **, P < 0.01, ***, P < 0.001 compared to naïve BMDM alone, Unpaired Student t test.

Apoptotic RPE cells were induced by exposing cells to 1mM H_2_O_2_ in serum-free DMEM overnight at 37°C [30]. Flow cytometry analysis confirmed that ~90% RPE cells were Annexin V^+^ or Annexin V^+^PI^+^ (Figure 5A). When BMDMs were incubated with apoptotic RPE cells, the expression of C3, CFB and C1INH was upregulated by 3.58-fold, 59.63-fold and 45.76-fold respectively, whereas the expression of C1q, CFH and CD59a was down-regulated to 0.20-fold, 0.03-fold and 0.55-fold (compared to BMDM alone) respectively (Figure 5B). The result suggests that apoptotic RPE cells may promote AP complement activation in subretinal macrophages.

### Changes in BMDM complement gene expression after culturing in RPE-choroid eyecup

**Figure 6:**
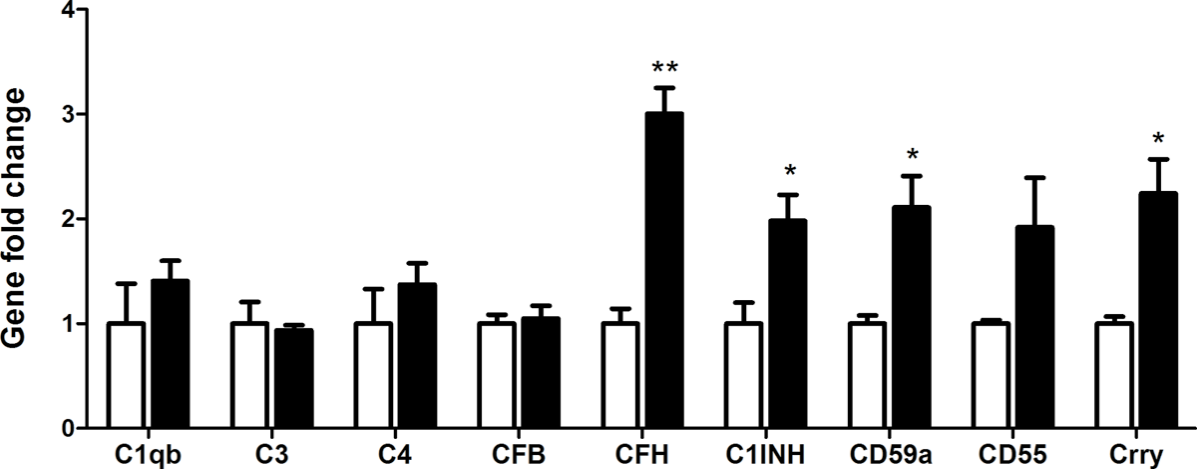
**The effects of RPE/choroid eye-cup on BMDM complement gene expression.** Naïve BMDMs were cultured in RPE/choroid eye cup from 18 months old mice for 7h. Macrophages were then isolated and processed for real-time RT-PCR analysis of complement genes. Mean ± SEM, n =3; *, P< 0.05; **, P < 0.01 compared to naïve BMDM alone, Unpaired Student t test.

To further understand how complement expression by subretinal macrophages is regulated by RPE cells in the aging eye, BMDMs were cultured in the eyecups of 18 months old mice. The cells were then removed from the eyecups and their complement gene expression was analyzed by real-time RT-PCR. Compared to naive BMDMs, the eyecup treated macrophages expressed significantly higher levels of CFH, C1INH, CD59a and Crry (2~3-fold increment) (Figure 6). There was a trend of upregulation in CD55 mRNA in eyecup treated macrophages (Figure 6, P = 0.12 respectively). The expression of other complement genes, including C1qb, C3, C4 and CFB remained unchanged (Figure 6). CFH and C1INH are secretory types of complement inhibitors, whereas CD59a, Crry and CD55 are membrane-type inhibitors. Our results suggest that subretinal macrophages are likely to suppress complement activation at the retina-choroid interface in the normal aging eye.

## DISCUSSION

RPE cells are known to critically control the immune suppressive microenvironment of the subretinal space through multiple mechanisms, including inducing the death of infiltrating immune cells (through the Fas Ligand and Tumor Necrosis Factor-related apoptosis-inducing ligand (TRAIL) [31–33]), releasing of immune suppressive mediators (e.g. TGF-β, retinoic acid, thrombospondin-1, CTLA4 and CLTA2) [34–37]). In this study, we show that RPE cells can modulate macrophage towards an anti-complement activation phenotype although inflammatory or apoptotic RPE cells may promote the alternative pathway of complement activation by inducing relevant complement expression in macrophages. The immune modulatory role of RPE cells on macrophage complement expression may be an additional mechanism to maintain retinal homeostasis particularly in the aging eye where macrophages accumulate in the subretinal space.

Previously we have reported a low-level of complement activation (e.g., increased CFB and C3/C3b expression) at the retina-choroid interface in the aging eye [11,14]. We believe that this low-level of complement activation is beneficial and important to maintain retinal homeostasis [14]. Uncontrolled complement activation is believed to be involved in the pathogenesis of AMD [4,10,38–40]. How complement activation at the immune privileged site of subretinal space (i.e., retina-choroid interface) is regulated and why the activation becomes detrimental in AMD is not known. Previous studies have observed higher plasma levels of C3a, C4a and C5a in AMD patients compared to age-matched controls [39–41]. However, a recent study by Schick et al did not find significant difference in the plasma levels of C3a, Ba, sC5b-9, CFH, CFI between AMD patients and age-matched controls, but the aqueous levels of C3a and Ba were significantly higher in AMD patients [42], suggesting that uncontrolled local but not systemic complement activation may play an important role in AMD pathogenesis.

RPE and subretinal macrophages are the only two types of cells in the retina-choroid interface in the aging eye, and they may critically control local complement activation. Previously, we have shown that RPE cells express high levels of complement regulators (e.g., C4BP, MASP1, CFH, C1INH, DAF, Crry and CD59a), but relative low levels of complement components (e.g., C2, C4, CFB and C5) [9,12]. This complement expression profile fits the immune suppressive nature of RPE cells and may contribute to the immune privilege of the subretinal space. Macrophages, on the other hand, express high levels of C1q under normal culture conditions [20]. C1q not only can initiate the classical pathway of complement activation, but also is critically involved in the phagocytosis of antibody-opsonized or FcR coated particles [26]. In this study, we show that normal RPE cultures are able to down-regulate the expression of C1qb and C3 and upregulate the expression of C1INH and CD59a in macrophages. The upregulation of complement regulators (CFH, CD59a, Crry, C1INH and CD55) was more prominent when macrophages were cultured in the RPE-choroid eyecups (Figure 6). Our results suggest that RPE cells may convert subretinal macrophages into a phenotype that can suppress complement activation. This “anti-complement activation” function of RPE may be a novel mechanism contributing to the immune suppressive microenvironment of the subretinal space.

The ability of RPE cells to regulate macrophage complement expression appears to rely on the physiological condition of the cells. After pre-treatment with oxPOS which induced RPE cells senescence, RPE cells further increased the expression of C1q and C1INH in macrophages (Figure 3). This phenotype allows macrophages to maintain complement suppressive property, but the phagocytosis function may be improved (as a result of increased C1q expression). oxPOS pre-treated RPE cells mimic the RPE cells in the aging eye [16], which may release debris into the subretinal space. This macrophage phenotype ensures effective clearance of the debris from the subretinal space to maintain homeostasis. Indeed, many subretinal macrophages in the aging eye contain pigmented inclusions (Figure 1B, 1C) [16].

Under disease conditions whereby RPE cells are subjected to inflammatory insult (e.g. TNF-α) or even undergo cell death, they may lose the immune suppressive property, instead become proinflammatory. TNF-α pre-treated RPE cells upregulated CFB and C1INH expression (Figure 4) in BMDMs, suggesting that they may suppress CP but promote AP complement activation. This macrophage phenotype was further enhanced by apoptotic RPE cells, evidenced by reduced expression of CP component (C1q) and AP inhibitor (CFH) and increased expression of CP inhibitor (C1INH) and AP components (CFB, C3) (Figure 5). This result suggests that under disease conditions, damaged or dead RPE cells may convert subretinal macrophages into a phenotype that can promote the AP complement activation at the retina-choroid interface. This concept is supported by our previous observation that retinal complement activation in autoimmune uveoretinitis is mediated predominately by the AP [15].

In summary, we show that RPE cells can modulate macrophage complement expression. Under normal aging conditions, RPE cells may convert macrophages into a phenotype that can suppress complement activation with enhanced phagocytosis. This immune regulatory function of RPE cells on macrophages may be lost under inflammatory conditions. Instead, inflammatory or apoptotic RPE cells promote macrophages to produce complement components necessary for the AP activation. RPE cells together with subretinal macrophages critically control complement activation at the retina-choroid interface in the ageing eye.

## MATERIALS AND METHODS

### Preparation of photoreceptor outer segments

Photoreceptor outer segments (POS) were isolated from bovine eyes using the sucrose gradient density centrifugation as previous described [43]. Oxidized POS (ox-POS) was generated by exposing POS to 302 nm ultraviolet light for 18 hours [43], and lipid oxidation was confirmed using thiobarbituric acid reactive substance assay (TBARS) kit (OXI-TEK TBARS Assay kit, Alexis; Axxora Ltd, Nottingham, UK). The levels of TBARS were confirmed to be 60 - 92 nmol/10^8^ POS in oxPOS and 5 ~ 11 nmol/10^8^ POS in non-oxidized POS.

### RPE cell culture

Primary mouse RPE cells were isolated and cultured from 3-month old wild type C56BL/6J mice as described previously [9,11]. Briefly, after removing the anterior segment of the eye, the vitreous and retina were peeled off from the eye cups. The RPE/choroid/sclera eye cups were incubated with 0.5% (w/v) trypsin-EDTA (ICN Flow, Irvin, UK) at 37°C for 30 min. RPE cells were harvested by gentle aspiration and the single-cell suspension was collected and seeded into culture plates with Dulbecco’s Modified Eagle Medium (DMEM), supplemented with 10% Fetal Calf Serum (FCS) and 100 mg/ml primocin. Cells were subcultured when they reached confluence. RPE phenotype was confirmed by their cytokeratin 19 expression. Cells of passage 3-5 were used for experiments.

To induce apoptosis in RPE cells, the cell cultures were treated with 1mM H_2_O_2_ in serum-free DMEM for overnight at 37°C [30]. Cells were detached by washing with ice-cold PBS and processed for Annexin V/Propidium Iodide (PI) staining according to manufacturer’s instructions (Life Technologies, UK), and examined by FACS Canto II (BD Biosciences, Oxford, UK).

To generate oxRPE cells or TNF-α pre-treated RPE cells, RPE cells were incubated with 1x 10^6^/ml ox-POS [43] or TNF-α (20 ng/ml) [11] for 24 h. Un-ingested POS or excessive TNF-α were then removed from the culture by thorough washes with warm PBS. RPE cells with different treatments were stained for β-Galactosidase using the Senescence β-Galactosidase Staining Kit (Sigma-Aldrich) as per manufacturer’s instructions.

### Culture of bone marrow-derived macrophages (BMDMs)

BMDMs were cultured using the protocol originally described by Weischenfeldt and Porse [44] with slight modification [20]. Briefly, tibias and femurs were collected from 8 to 12-week old wild-type C57BL/6J mice. Bone marrow was flushed with DMEM (Gibco BRL, Paisley, UK). Red Blood Cells (RBCs) were lysed by RBC Lysis Buffer (0.75% NH_4_Cl, 0.02% Tris-HCl, pH 7.2). Cells were then washed and cultured in DMEM supplemented with 15% FCS and 15% L929 (a murine fibroblast cell line that secretes M-CSF) conditioned medium containing 100 mg/ml primocin (Invivogen, San Diego, California, USA) in 75 cm^2^ Culture Flasks, and incubated at 37°C in 5% CO_2_ incubator. After 6 days, cells were collected for further experiments. Flow cytometry confirmed that >92% of the cells were F4/80^+^CD11b^+^.

### BMDM-RPE co-culture

1.5 x 10^6^ BMDMs were seeded into T25 flask RPE cultures (5 x 10^5^ of normal RPE, or ox-RPE or TNF-α-RPE or apoptotic RPE cells) and co-cultured for 7h or 24h. Cells were then detached in ice-cold PBS with 2mM EDTA and passed through 70 μm cell strainer. BMDMs were isolated with the CD11b^+^ MACS kit (Miltenyi Biotec, UK). The phenotype of the BMDMs was confirmed to be 95-98% CD11b^+^F4/80^+^ by flow cytometry (FACS Canto, BD Biosciences, UK). The cells were processed further for downstream experiments.

### Macrophage culture in the eye-cup

Eyes from 18 months old C57BL/6J mice were sterilized with 75% ethanol and dissected under microscope. The anterior segment of the eye, the lens and retinal tissue were removed. The eyecups containing RPE, choroid and sclera were used for further studies.

1 x 10^4^ BMDMs were added into the eyecup and cultured at 37 °C in 5% CO2 incubator for 7h. BMDMs on eyecup were harvested by gently shaking the eyecup in a tube with culture medium. 90-95% of cells were F4/80^+^ by flow cytometry. The cells were collected and processed for real-time RT-PCR.

### RNA isolation and reverse transcription

Total RNA was extracted from cultured BMDMs using the RNeasy mini kit (Qiagen Ltd., West Sussex, UK) according to the manufacturer’s instructions. The quantity and quality of the RNA were determined using the NanoDrop ND-1000 spectrophotometer (NanoDrop Technologies, Wilmington, DE). First-strand cDNA synthesis was performed by a reaction of 1 µg of total RNA with a random primer, using the SuperScript^TM^ II Reverse Transcriptase kit (Invitrogen, Paisley, UK).

### Real-time Polymerase Chain Reaction (Real-time RT-PCR)

Real-time RT-PCR was performed in a total of 12 µl mixture solution in 384-well plates using the LightCycler 480 system (Roche Applied Science, Mannheim, Germany). Each 12 µl of reaction mixture contains 6 ml of LightCycler 480 SYBR Green Master (Roche Diagnostics GmbH, Mannheim, Germany), 0.5 mM primers and diluted cDNA. Real-time RT-PCR quantifications were run in triplicate for each sample and the average determined. PCR products were quantified by the LightCycler 480 software. Melting curve and gel electrophoretic analyses were used to determine amplification homogeneity and data quality. Expression levels were normalized to β-actin. The primer sequences have been described previously [12,20].

### Western blot

BMDMs were lysed in RIPA buffer with protease inhibitors (Sigma Aldrich, USA). The total protein concentration was measured using a BCA kit (Solarbio, China). Ten micrograms of total protein were applied to 10% SDS-PAGE and blotted to an Immobilon-FL polyvinylidene difluoride membrane (Millipore, China). After blocking with 5% fat-free milk, the membrane was incubated with primary antibodies for C1INH (Abcam, UK) and β-actin (Arigo Biolaboratories, China) at 4 °C overnight, followed by incubation with secondary antibodies respectively at room temperature for 1-2 h. Quantitative imaging was performed using Odyssey infrared imaging system (Li-COR Biotechnology, USA). Protein bands were quantified using ImageJ software.

### Data analysis

All data were expressed of Mean ± Standard error of mean (SEM). The difference in complement gene expression levels between normal un-treated BMDMs and different types of RPE treated BMDMs were compared using the unpaired Student t test with P < 0.05 considered to be statistically significant. All experiments were repeated at least twice.
